# Harnessing technology to enable all women mobility in labour and birth: feasibility of implementing beltless non-invasive fetal ECG applying the NASSS framework

**DOI:** 10.1186/s40814-021-00953-6

**Published:** 2021-12-07

**Authors:** Deborah Fox, Rebecca Coddington, Vanessa Scarf, Andrew Bisits, Anne Lainchbury, Rachael Woodworth, Robyn Maude, Maralyn Foureur, Jane Sandall

**Affiliations:** 1 Centre for Midwifery Child and Family Health, University of Technology Sydney, Sydney, Australia; 2 Royal Hospital for Women, NSW Health, Sydney, Australia; 3 Victoria University of Wellington, Wellington, New Zealand; 4 University of Newcastle, Newcastle, NSW Australia; 5 King’s College London, London, UK

**Keywords:** Midwifery, Obstetrics, Intrapartum, Labour and birth, Freedom of movement, Fetal monitoring, NASSS, Non-invasive fetal ECG (NIFECG), Transabdominal fetal monitor

## Abstract

**Background:**

A new wireless and beltless monitoring device utilising fetal and maternal electrocardiography (ECG) and uterine electromyography, known as ‘non-invasive fetal ECG’ (NIFECG) was registered for clinical use in Australia in 2018. The safety and reliability of NIFECG has been demonstrated in controlled settings for short periods during labour. As far as we are aware, at the time our study commenced, this was globally the first trial of such a device in an authentic clinical setting for the entire duration of a woman’s labour.

**Methods:**

This study aimed to assess the feasibility of using NIFECG fetal monitoring for women undergoing continuous electronic fetal monitoring during labour and birth. Women were eligible to participate in the study if they were at 36 weeks gestation or greater with a singleton pregnancy, planning to give birth vaginally and with obstetric indications as per local protocol (NSW Health Fetal Heart Rate Monitoring Guideline GL2018_025. 2018) for continuous intrapartum fetal monitoring. Written informed consent was received from participating women in antenatal clinic prior to the onset of labour. This single site clinical feasibility study took place between January and July 2020 at the Royal Hospital for Women in Sydney, Australia.

Quantitative and qualitative data were collected to inform the analysis of results using the NASSS (Non-adoption, Abandonment, Scale up, Spread and Sustainability) framework, a validated tool for analysing the implementation of new health technologies into clinical settings.

**Results:**

Women responded positively about the comfort and freedom of movement afforded by the NIFECG. Midwives reported that when no loss of contact occurred, the device enabled them to focus less on the technology and more on supporting women’s physical and emotional needs during labour. Midwives and obstetricians noticed the benefits for women but expressed a need for greater certainty about the reliability of the signal.

**Conclusion:**

The NIFECG device enables freedom of movement and positioning for labouring women and was well received by women and the majority of clinicians. Whilst measurement of the uterine activity was reliable, there was uncertainty for clinicians in relation to loss of contact of the fetal heart rate. If this can be ameliorated the device shows potential to be used as routinely as cardiotocography (CTG) for fetal monitoring. This is the first time the NASSS framework has been used to synthesise the implementation needs of a health technology in the care of women during labour and birth. Our findings contribute new knowledge about the determinants for implementation of a complex technology in a maternity care setting.

**Trial registration:**

The Universal Trial Number is reU1111-1228-9845 and the Australian and New Zealand Clinical Trial Registration Number is 12619000293167p. Trial registration occurred on the 20 February, 2019. The trial protocol may be viewed at http://www.anzctr.org.au/Trial/Registration/TrialReview.aspx?id=377027

**Supplementary Information:**

The online version contains supplementary material available at 10.1186/s40814-021-00953-6.

## Key messages

- What are the key feasibility findings?

The majority of stakeholders were optimistic about the potential for the NIFECG device to be developed and up-scaled in practice. Women who were interviewed described the comfort afforded by the beltless design and lightweight nature of the NIFECG device. Freedom of movement and positioning was enabled for women after an initial calibration period of 15 min; hence, the device makes a significant contribution to optimising bodily autonomy and physiological processes in labour for women undergoing continuous intrapartum fetal monitoring.

- What uncertainties existed regarding the feasibility?

Whilst the reliability of the electromyography in measuring uterine activity was relatively good (83% of cases), there was uncertainty for clinicians in relation to loss of contact with the fetal heart rate. In 27.3% of cases, midwives were unable to get all three signals (fetal heart, maternal heart and uterine activity) to work, despite troubleshooting for 30–90 min. In 31% of cases (*n* = 34), the device worked well for a length of time but was later discontinued due to malfunction or sudden loss of contact.

- What are the implications of the feasibility findings for the design of the main study?

The feasibility findings indicate that further work is needed to refine the technology in relation to preventing unexpected loss of contact, especially in the second stage of labour.

This feasibility study was conducted in an urban setting that has low rates of women who are overweight or obese. Due to the existing experimental evidence around the increased efficacy of NIFECG over the CTG in women with a high body mass index (BMI), further research is needed in this cohort in the Australian context. This is important because overweight and obesity affects approximately half the population of women of childbearing age (18–44 years of age) in Australia [1].

The design of the main study will incorporate an exploration of barriers and facilitators to implementation in different settings, particularly regional, rural and remote contexts.

## Background

A variety of methods are used in clinical practice to monitor fetal well-being during childbirth. These include intermittent auscultation (IA) of the fetal heart, using a Pinard fetoscope or handheld Doppler, and technologies that enable the fetal heart and uterine activity to be measured continuously. Since the introduction of wired cardiotocography (CTG) in the 1960s, continuous measurement of the fetal heart has commonly been performed with equipment that requires the labouring woman to wear two elastic belts around her abdomen and to be tethered to a machine by wiring. This technology restricts women’s mobility during labour and limits their choice of position whilst giving birth.

Evidence demonstrates that freedom of movement and positioning during childbirth results in a shorter duration of labour, lower likelihood of caesarean section and fewer epidurals, and is not associated with any negative effects for women or their babies [2–4]. Furthermore, freedom to move has psychological benefits because it strengthens women’s sense of choice and control during their birth experience [4, 5]. Choice and control are important to women in childbearing [2, 4–6]. When women feel that they lack choice and control in labour, levels of stress and pain perception are elevated, resulting in increased need for pharmacological pain management [2].

International clinical guidelines recommend that continuous electronic fetal monitoring (EFM) should only be offered to women identified as having complex pregnancies or as being at high risk of fetal complications in labour [7–9]. Of approximately 300,000 women who give birth each year in Australia [10], we estimate that more than half experience continuous CTG monitoring for indications including previous caesarean section, multiple pregnancy, induction/augmentation of labour, epidural analgesia and/or delayed progress in labour [11]. Because many women with complex pregnancies and/or risk factors are known to experience increased feelings of stress and vulnerability [12], it is desirable to provide fetal monitoring technologies that optimise physiological processes, strengthen women’s capabilities and increase their sense of choice and control.

To date, studies evaluating the efficacy of continuous monitoring have been conducted primarily on conventional wired technologies that restrict women’s movement and positioning [13, 14]. Wireless CTG that enables freedom of movement emerged in 2003, however, evidence from surveys conducted in the United Kingdom (UK) [15] and Australia and New Zealand [16] demonstrate that it is still not being used routinely in many hospitals. Findings from both surveys indicated that the majority of women being continuously monitored are still receiving wired technology [15, 16]. In many settings, wireless technology that enables freedom of movement in labour has been purchased and is potentially situated in the birth unit but is not routinely accessible or available to women. These phenomena are currently being explored by our team via further in-depth qualitative research exploring the barriers and facilitators for midwives in implementing use of wireless CTG.

A new wireless and beltless monitoring device utilising fetal and maternal electrocardiography (ECG) and uterine electromyography (EMG) was released to the Australian market in 2019. In the majority of the literature, this form of fetal monitoring technology is referred to as ‘non-invasive fetal ECG’ (NIFECG) [17–19]. The safety and reliability of NIFECG is demonstrated in the results of experimental research conducted in a range of settings including the UK, USA, Europe and Israel [20–28]. Furthermore, NIFECG has been shown to be more reliable than conventional CTG monitoring for women with a BMI of 30 kg/m^2^ or above [21, 29]. None of these studies have explored the use of NIFECG for the entirety of labour and birth. As such, this is the first feasibility study we are aware of that explores the use of NIFECG device throughout labour.

The alternative to external EFM is the fetal scalp electrode (FSE), which is often employed in the care of women with a high BMI or when there is difficulty maintaining a reliable fetal heart trace. Whilst FSE is more reliable than external CTG in recording the fetal heart rate, it is invasive to both mother and baby. FSE requires artificial rupturing of maternal membranes and puncturing of the skin surface of the fetal scalp. Due to the limitation that FSE does not measure contractions, it does not provide a complete assessment of fetal heart rate patterns in relation to the frequency and timing of uterine contractions. Anecdotally, in Australia, we have seen an increase in the use of intrauterine pressure catheters (IUPC) in women with obesity as this is thought to provide a more reliable reading of uterine contractions. However, similar to the FSE, the IUPC is invasive for the woman, requires artificial rupturing of her membranes and places women at increased risk of infection. NIFECG technology has the potential to replace or reduce the use of FSE and IUPC in obese populations. This is significant because almost half (47.5%) of the 298,567 women giving birth in Australia in 2019 were overweight or obese [10].

In 2018, a NIFECG device known as the Philips Avalon Beltless Solution (PABS) was registered by the Therapeutic Goods Administration (TGA) for clinical use in Australia. Further details about the Philips Avalon Beltless Solution and an image may be found in the Additional file 1.

This study aimed to assess the feasibility and acceptability of using the new beltless and wireless NIFECG fetal monitoring device (PABS) that enables mobility for women undergoing continuous EFM during labour. The results of this feasibility study will inform the design of a hybrid randomised controlled trial comparing different forms of intrapartum fetal monitoring for women with increased BMI and future studies investigating the association between mobility in labour and outcomes for women being continuously monitored. As far as we are aware, at the time our study commenced, this was the first trial of the device in an authentic clinical setting for the duration of a woman’s labour.

## Methods

### Study design

This feasibility study synthesised both quantitative and qualitative data to inform an analysis using the Non-adoption, Abandonment, Scale up, Spread and Sustainability (NASSS) framework [30], a validated tool for analysing the implementation of new health technologies into clinical settings. This framework may be used to identify and assess barriers and facilitators for future large-scale implementation of health technology innovations [30]. The NASSS framework was chosen as it is a theoretical framework consisting of seven domains which, when used to appraise the different facets of the implementation of a health technology, allows researchers to appraise the complexity of the process of implementation. The NASSS framework will be explained in more detail in the data analysis section.

### Context and clinical setting

This single site clinical feasibility study took place between January and July 2020 in the Royal Hospital for Women (RHW) in Sydney, Australia. This is a public teaching hospital facility which provides maternity, neonatal, gynaecology and oncology services. RHW has more than 4200 births per year, receives referrals for neonatal specialty care from throughout the state of New South Wales and is part of a network which caters for the highest level of acuity. It also offers models of midwifery continuity of care for women of all risk categories.

### Participants

#### Women

Women were eligible to participate in the study if they were at 36 weeks gestation or greater with a singleton pregnancy, planning to give birth vaginally and with obstetric indications as per local protocol [11] for continuous intrapartum fetal monitoring. We aimed to recruit 100 women for the clinical component of the study, which required women to consent to wearing the NIFECG (PABS) device to monitor the well-being of their fetus during labour. At the time of recruitment into the clinical component of the study, women were also invited to consent to being contacted by telephone 4–6 weeks after giving birth, for a semi-structured telephone interview exploring their views and experiences. We aimed to recruit 20 women for interview. Women who declined to be contacted for an interview were not excluded from the clinical component of the trial.

#### Midwives and obstetricians

Midwives and obstetricians who cared for women participating in the clinical component of the study were invited to take part in pre and/or post-intervention interviews or focus groups. The inclusion criteria for midwives’ participation in the pre-intervention focus group was that they were an employee at the clinical site and had provided direct clinical care to labouring women in the birth unit. The criteria for clinicians’ participation in the post-intervention interviews and focus group was their involvement in the direct clinical care of at least one labouring woman trialling the device (PABS) during labour.

### Data collection

The primary outcome of interest was the feasibility of using a new beltless and wireless intrapartum fetal monitoring device (PABS) that enables freedom of movement for women with clinical indications for continuous monitoring in an Australian clinical setting. Women were recruited in the antenatal clinic or antenatal ward and the study was conducted in the birth unit.

Quantitative data collection included gathering routinely collected clinical data from the medical records of participating women and inspection of midwifery and medical records and fetal monitoring traces of the participating women. These data were synthesised with the results of brief questionnaires distributed to midwives caring for participating women. The brief, three-question questionnaire consisted of asking midwives to record (a) each participating woman’s birth position and (b) the reason for ceasing use of the device, if this occurred and (c) the mode of monitoring used for the remainder of the woman’s labour.

The secondary outcomes data included routinely collected clinical outcomes for labouring women participating in the trial. These outcomes included onset of labour and mode of birth, length of labour, use of analgesia, fetal blood sampling, perineal outcomes, Apgar score, NICU admission over 48 h and length of hospital stay. Although this feasibility study was not powered to detect changes in clinical outcomes, clinical data were used to identify any preliminary trends that may warrant further investigation in future research. Aggregated data on outcomes will be made available on request from the first author. We are unable to publish the data freely as the sample is small and derived from one site, thereby presenting a risk to participants’ confidentiality.

Qualitative data explored the views and experiences of the women, midwives and obstetricians involved, using focus groups and semi-structured interviews. These data informed the analysis, using the NASSS framework, of the acceptability, usability and stakeholder perceptions around future adoption of the device. The views and experiences of 15 women, 22 midwives and 5 obstetricians on the clinical use of the device were explored, aiming to focus on the impact of the device upon women’s mobility in labour. Focus groups with clinicians were held prior to the commencement of the use of the device (PABS) and at the completion of the quantitative data collection. Focus groups were led by two members of the research team.

Five obstetricians employed at the site who had the experience of caring for women monitored with the NIFECG device (PABS) participated in semi-structured telephone interviews about their experiences of caring for women using the device.

The purpose of the interviews with women was to explore their views and experiences of being monitored during labour with the beltless device. The primary exposure of interest was mobility in labour. All interviews were audio recorded and immediately transcribed for analysis.

### Data analysis and storage

Multiple sources of data, including medical records, questionnaires, routinely collected data and inspection of the fetal monitoring traces, were synthesised and analysed using the NASSS framework. The NASSS framework enables the analysis of seven domains of relevance to implementation: the condition, the technology, the value proposition, the adopters, the organisation, the wider system and embedding and adaptation over time.

All data were stored on a secure cloud-based institutional system at the University of Technology Sydney (UTS). Only appropriate team members were granted permission to access raw data. Hard copies of consent forms were stored at UTS in a locked cabinet in the locked office of the chief investigator (DF). Pseudonyms were used to de-identify qualitative data and to protect the privacy and confidentiality of participants. After 15 years, data will be destroyed, in accordance with the Australian Code for the Responsible Conduct of Research [31].

### Ethical considerations

Ethics approval to conduct the study was received from the Human Research Ethics Committee (HREC) of NSW Health South East Sydney Local Health District at the clinical site (Ethics Approval Number 2019/ETH00630) and ratified by the University of Technology Sydney HREC (Ethics Approval Number ETH19-3744).

All participants were given written and verbal information about the device and the study prior to providing written informed consent and were free to withdraw at any time. Participants were informed that their identity would be kept confidential and all transcripts of interviews and focus groups were re-identified using pseudonyms.

The NIFECG (PABS) device is TGA approved for clinical use in Australia. The Universal Trial Number is reU1111-1228-9845 and the Australian and New Zealand Clinical Trial Registration Number is 12619000293167p. Reporting of the study complies with the CONSORT Extension for non-randomised pilot and feasibility studies.

This study was an investigator designed and led project, sponsored by the University of Technology Sydney and industry funded by Philips Medizin Systeme Germany. The funder had no role or influence in the design of the study, collection of data, analysis of data, interpretation of data or writing the manuscript.

## Results and discussion

**Fig. 1:**
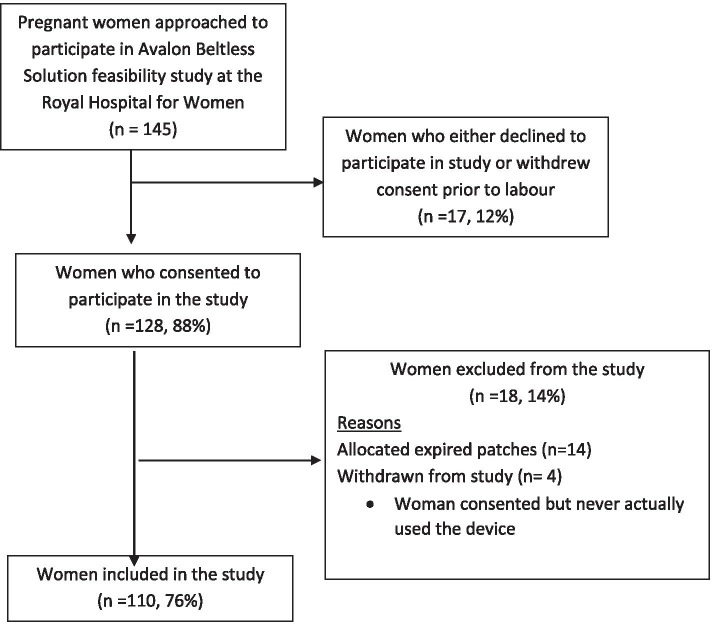
Flowchart of the recruitment and inclusion process

In total, 145 eligible women were approached in the antenatal clinic by the clinical midwifery consultant (CMC) and/or research midwife and invited to participate in the clinical component of the study. Informed consent to participate was given by 128 pregnant women who met the inclusion criteria and 110 women completed the trial (see Fig. 1).

Data from the midwives’ questionnaires provided information regarding each woman’s position at birth and any reason why the NIFECG (PABS) was discontinued. Researchers augmented these data by referring to the medical records and fetal monitoring traces.

Of the 110 women who participated in this study, 72% were nulliparous and the majority were aged between 30 and 40 years. Table 1 illustrates the demographic characteristics of the included women and their outcomes related to the birth. Seven women had a gestation of 36–37 completed weeks and 57% of women had a gestation over 40 weeks. The majority of women had an induced onset of labour (84.5%); this was in part due to the need to recruit women who knew during late pregnancy that they were consenting to continuous EFM in labour. The caesarean section rate was 28.2%, and 71.8% of women had a vaginal birth (normal vaginal birth 48.2%, instrumental birth 23.6%). Most women used some form of analgesia and many women used more than one type, with 75 women (68.2%) having an epidural or spinal block. The majority of babies had an Apgar score over seven (98.2%) at 5 min, and no baby required active resuscitation.

**Table 1:** Demographic details and maternal and perinatal outcomes

	*N* (%)
**Maternal age (years)**	
**Range (mean)**	19-47 (33.7)
**19–25**	9 (8.2)
**> 25–30**	16 (14.5)
**> 30–35**	44 (40)
**> 35–40**	31 (28.2)
**> 40**	10 (9.1)
**Previous pregnancies (> 20 weeks)**	
**0**	80 (72.7)
**1**	24 (21.8)
**2**	4 (3.6)
**≥ 3**	2 (1.8)
**Gestation (weeks)**	
**≤ 37**	7 (6.4)
**38**	13 (11.8)
**39**	27 (24.5)
**≥ 40**	63 (57.3)
**BMI**	
**< 30**	99 (90)
**> 30**	11 (10)
**Onset of labour**	
**Spontaneous**	7 (6.4)
**Induction of labour**	103 (84.5)
**Epidural/spinal analgesia**	
**Yes**	75 (68.2)
**No**	35 (31.8)
**Length of labour (hours)**	
**Minimum**	20 min
**Maximum**	11.6 h
**Mean**	4.5 h
**Mode of birth**	
**Normal vaginal birth**	53 (48.2)
**Instrumental birth**	26 (23.6)
**Caesarean birth**	31 (28.2)
**Perineal outcomes**^b^	
**Intact perineum**	6 (5.5)
**Graze/first degree tear**	9 (8.2)
**Second degree tear**	27 (24.5)
**Third degree tear**	6 (5.5)
**Episiotomy**	31 (28.2)
**Length of stay (days)**	
**0**	11 (10.0)
**1**	30 (27.3)
**2**	31 (28.2)
**3**	21 (19.1)
**4**	10 (9.1)
**≥ 5**	7 (6.3)
**Fetal blood sampling (FBS)**	
**Yes**	33^a^
**No**	85
**Fetal scalp electrode use**	
**Yes**	11 (10)
**No**	99 (90)
**Apgar score at 5 min**	
**< 7**	2
**≥ 7**	108

^a^There were 33 FBS in total with a range of 1-3 samples per fetus

^b^Only vaginal births included here

Although this feasibility study was not powered to detect changes in clinical outcomes, clinical data were used to identify any preliminary trends that may warrant further investigation in future research. No significant trends were identified in comparison to overall hospital data, apart from low incidence of fetal scalp electrode use (10% overall). It was particularly notable that of 11 participating women classified as obese, only one woman required a fetal scalp electrode to measure fetal heart rate. This supports findings in the existing literature surrounding the efficacy of the NIFECG (PABS) in women with increased BMI and will be explored in future research.

The reliability of the signal of the PABS in relation to the observable trace was recorded in part on the questionnaires completed by the midwives and data collected from the traces and medical notes. The device was unsuccessful for 27.3% of women (*n* = 30) due to reasons such as an inability to calibrate the machine from the beginning of the monitoring period or an inability to connect all electrodes to the receiver on the base machine (signal issue). The remainder of women had successful monitoring for part of the first stage of labour (30%, *n* = 33), successful monitoring for the entire first stage then loss of contact/signal occurred (19.1%, *n* = 21) and 23.6% of women (*n* = 26) had their labour monitored successfully until the birth of the baby (Table 2). There were some women for whom the NIFECG (PABS) was working successfully (*n* = 27) but discontinued for reasons such as transfer to operating theatre (*n* = 10), request to use the bath, transcutaneous electric nerve stimulation (TENS) or a Bluetooth device (*n* = 7), clinician request due to concern about fetal wellbeing (*n* = 7), or other reason (*n* = 3).

**Table 2:** Reliability of the NIFECG (PABS)

	***N***	%
**All women (*n*** **= 110)**		
**Unsuccessful**^a^	30	27
**Successful use throughout 1st stage of labour**	21	19
**Successful use throughout part of the 1st stage of labour**	33	30
**Successful use throughout the entire labour and birth**	26	24
**Total**	110	100
Nulliparous women (*n* = 80)		
Unsuccessful^a^	26	32
Successful use throughout 1st stage of labour	14	18
Successful use throughout part of the 1st stage of labour	30	37
Successful use throughout the entire labour and birth	10	13
Total	80	100
Multiparous women (*n* = 30)		
Unsuccessful^a^	4	13
Successful use throughout 1st stage of labour	7	23
Successful use throughout part of the 1st stage of labour	3	10
Successful use throughout the entire labour and birth (1st & 2nd stage labour)	16	54
Total	30	100

^a^Unsuccessful use is defined as attempting to achieve a connection for between 30 and 90 min without success, despite troubleshooting

There were eleven participants (10%) with a BMI > 30. The device worked successfully throughout labour for eight of these women. Of the remaining three women, there were problems picking up contractions via EMG for one, fetal and maternal rate crossover resulting in malfunction for one and one for whom the device completely stopped working for unknown reasons.

Qualitative data were collected via focus groups and interviews with 22 midwives, 5 obstetricians and 15 women. Below, we apply the different domains of the NASSS framework to the findings from both the quantitative and qualitative components of our study.

### Domain 1: The condition of childbirth

Childbirth is a highly complex process that is impacted by multiple clinical, psychosocial and environmental factors. Labour is not linear; it is complex, influenced by a multitude of factors and often unpredictable [32]. Clinicians and caregivers are mindful that there are two ‘patients’ to consider simultaneously, the woman and her baby. Despite the clear legal and human rights perspectives that a woman has the same autonomy over her body whether or not she is pregnant, there exist competing views on whose needs are to be prioritised, the woman or the baby. Despite a universal agreement on the need for care that results in a ‘healthy mother and a healthy baby’, there are conflicting views on how that care is best delivered, stemming from conflicting professional and epistemological paradigms around maternity care [33].

Evidence demonstrates that mobility during labour and freedom of positioning in birth results in a shorter duration of labour, lower likelihood of caesarean section and fewer epidurals [2–4]. However, since the introduction of wired CTG in the 1960s, continuous measurement of the fetal heart has commonly been performed with equipment that requires the labouring woman to wear two elastic belts around her abdomen and to be connected to a machine by wiring. This technology restricts women’s mobility during labour and limits their choice of position whilst giving birth. International clinical guidelines recommend that continuous EFM should only be offered to women identified as having complex pregnancies or at high risk of fetal complications in labour [7–9].

#### Childbirth for women with complex pregnancies

Because many women with complex pregnancies are known to experience increased feelings of stress and vulnerability [12], it is desirable to provide woman centred care and fetal monitoring technologies that optimise physiological processes, strengthen women’s capabilities and increase their sense of choice and control. Hence, the need for the innovation includes the demand from women for freedom of movement during labour, choice of position when giving birth and improved comfort, whilst maintaining an effective mode of monitoring fetal wellbeing.

The demand from midwives is for a non-invasive technology that enables them to focus on caring for the woman, whilst maintaining reassurance about fetal wellbeing. Midwives seek technology that supports the woman’s bodily autonomy during labour and assists clinical decision making without compromising the physiological processes of labour and birth.

Obstetricians and midwives need certainty and confidence that the technology is reliable and provides effective data representing fetal wellbeing and that it will assist in the identification of the ‘at risk’ fetus.

The condition, childbirth, is clearly a complex domain as it is unpredictable and strongly influenced by external factors such as perceptions of risk and safety, the woman’s comorbidities as well as social and cultural factors. In addition, complexity is increased by the presence of two entities, the woman and the fetus. Furthermore, for women with complex pregnancies, there may be multiple care providers involved who are influenced by differing paradigms of childbearing. This may include, for example, the woman’s partner, a midwife, an obstetrician, an anaesthetist and/or a paediatrician.

### Domain 2: The technology

The routine use of wired CTG is problematic because it restricts the bodily autonomy of labouring women during, arguably, one of the most vulnerable experiences in their lives. Metre long wires attached between the transducers placed on a woman’s abdomen and the CTG machine mean that the woman is tethered to the machine during her labour.

Wireless CTG or ‘telemetry’ has been available since 2003; however, like conventional wired CTG, the wireless CTG relies on the positioning of transducers on the woman’s abdomen via the use of two tight elastic belts. Women we interviewed who had used CTG in the past reported that they found the belts uncomfortable. Midwives who participated told us that the use of wireless CTG is hindered by frequent hands-on adjustments that are required to keep the device operating reliably whilst the woman is mobilising.

The design of this NIFECG (PABS) is woman centred, in that it is wireless, beltless, lightweight and comfortable for women to wear. More detail on women’s views is included in the “Domain 4: The adopters” section.

The results demonstrated that for 73% of women (*n* = 80), the device worked well for all or part of the woman’s labour and birth. For 27% of women (*n* = 30), midwives were unsuccessful in initiating use of the device after application of the patch. Unsuccessful initiation is defined as attempting to achieve a connection to the monitor and calibrating the pod for at least 30 min, despite troubleshooting. Thirty-six women (33%) used the device successfully until the birth of their baby (either vaginally or by caesarean section). When use of the device was discontinued (40%), it was for two main reasons; maternal request (*n* = 10) and technology malfunction (*n* = 34). Reasons given for maternal request include water immersion in the bath, use of TENS for pain relief, skin irritation and the use of a Bluetooth medical device. Discontinuation due to technical malfunction included loss of connection to the monitor, the loss of interpretable trace despite attempts at regaining connection and loss of contact that often occurred when maternal and fetal heart rates were similar. In many instances, CTG monitoring replaced the NIFECG (PABS); however, upon review of the traces, we noted that the CTG trace contained similar loss of interpretability. We hypothesise that discontinuation in these scenarios may have been due to clinician anxiety as a result of lack of familiarity with the device and stress caused by detection of abnormal FHR pattern.

The technology is suitable for specific cohorts of women. When the pod is first applied, women are required to sit still for a period of 15 min to allow the machine to calibrate. As such, it is most suited to women who are in early labour or experiencing induction of labour. Although suitable for showering, the beltless device is not designed for use during water immersion (bath or birth pool) or in conjunction with TENS devices or external Bluetooth devices such as personal speakers for music. Despite this recommendation from the manufacturers, a number of women did use it successfully with TENS and/or Bluetooth during our study. It is also unsuitable for women who are allergic to medical adhesives due to potential skin irritation. Very few women experienced minor skin irritations following use; however, all but one indicated that it would not prevent them from using the device again in future.

For clinicians, the NIFECG device (PABS) is compatible with existing technologies, reducing uncertainty for users. The visual display of data and methods of interpretation are the same as existing EFM technologies. The device is fully integrated with the Philips Avalon CTG docking station and the existing technology remains accessible if needed. The use of a familiar and trusted fetal monitoring brand was thought to have reduced uncertainty for clinicians.

The cost implications for the hospital include software upgrades, purchase of pods and single-use patches. However, there is an anticipated reduction in cost associated with single-use belts, ultrasound gel and fetal scalp electrodes. Future research may determine whether there is further reduction in cost due to decreased rates of intervention such as epidural and caesarean section that may occur due to women having freedom of movement and positioning in labour and birth.

The technology domain is complicated, due to the kind of data generated and high levels of clinical disagreement that can occur on what the data mean. However, there are a number of elements of the NIFECG technology that resemble the CTG, such as the way in which data are displayed and methods used for interpretation. This lowers the complexity for clinicians in adopting the NIFECG.

Modifiable factors such as the need for more reliability in continuously collecting data on the fetal heart rate were found to exist. Once these technical issues are ameliorated, the NIFECG device shows potential to be used as routinely as cardiotocography (CTG) for fetal monitoring.

### Domain 3: Value proposition

#### Desirability for women

Women enjoyed freedom of movement when using the PABS device. The beltless design allowed for the adoption of numerous positions during labour and when giving birth and gave women the opportunity to access the bathroom without asking for permission or assistance from a midwife. Women reported an increased sense of control in labour when using the PABS device. Evidence suggests that upright positions in labour result in a shorter duration of labour, less epidurals, less caesarean section [3] and higher maternal satisfaction [4]. For women who did choose to use an epidural block, there was no interruption to continuous EFM and the beltless design was comfortable and convenient.

When being cared for in a universal healthcare setting such as Australia, there is no individual cost to the woman when using this device. Given that the use of the PABS device enabled freedom of movement and bodily autonomy for women, it has the potential to reduce intervention which provides short and long term benefits to women and babies, along with cost savings for health systems.

#### Benefits for the organisation

The majority of midwives felt the device enabled an improvement in quality of care provided to women. This was due to the reduction in the need to readjust the fetal monitoring device allowing the midwife to spend more time focussed on the woman and less time focussed on the technology. The device allowed for continuation of monitoring during epidural insertion, potentially increasing safety for women and their babies in the event of fetal distress.

#### Supply side value

This device has the potential to become a game changer in the way fetal monitoring is conducted during labour and birth, which would be undoubtedly profitable for the manufacturer. The device is already registered for therapeutic use with the TGA. A selling point is that the device is compatible with the existing technology used in 80% of hospitals in Australia. The technology brand is trusted throughout healthcare institutions globally. With potential to become a superior product, particularly for women who are difficult to monitor with CTG, the future market potential is significant.

### Domain 4: The adopters

The adopters who participated in this feasibility study were women, midwives and obstetricians in a public maternity hospital in an urban area of Sydney NSW, Australia. There was enthusiastic support from clinicians at the study site for trialling the device. Support included all levels of the organisation ranging from senior and middle management, to obstetricians and midwives and the women enrolling to participate. Integral to the success of the feasibility study was the enthusiasm of the organisation’s most senior obstetrician and lead clinical midwifery consultant (CMC), who championed the project and provided practical support to the staff using the device. The CMC also acted as a conduit between the research team and the study site, communicating regularly on the progress of the study and the collection of data. Information sessions for midwives and obstetricians were held in February and July 2019 at which approval to participate was gained from staff, who also provided feedback on the proposed study design.

A midwife’s responsibility caring for a woman during labour in an Australian public hospital context such as the clinical study site involves providing clinical care and documentation, emotional and physical support, monitoring the health and wellbeing of the woman and baby, optimising the woman’s physiological processes and physical comfort and referring to medical care if needed. During the care of a woman in labour who is undergoing continuous fetal monitoring, midwives also carry the primary responsibility for ensuring that the technology is applied correctly, functioning well and that a readable trace is being produced. Midwives are responsible for aiming to establish and maintain a good quality trace whilst also trying to support the woman’s birth plan which may have altered due to need for continuous monitoring. The aim is for all clinicians to be reassured that the visual data are an accurate reflection of the well-being of the fetus. When the trace is not reassuring, clinical decisions need to be made by obstetricians as to whether more invasive testing or intervention is required. Hence, obstetricians focus on the quality of the trace and on whether the signals being produced are demonstrating any signs of an ‘at risk’ fetus.

Most midwives readily adopted the new device and found it easy to integrate into their usual care of women. The midwives had to adopt a new way of applying the fetal monitoring device. This included washing and drying the skin on the woman’s abdomen, exfoliating the skin, applying the patch and pod and calibrating the pod with the base station machine. Midwives at the study site were committed to routinely supporting women to mobilise and use upright positions in labour. As such, the beltless device was keenly anticipated to enhance women’s capacity to mobilise during labour. Further to this, the interpretation of visual data was no different from standard care. Administrative staff were not impacted in any way.

There were significant technical difficulties encountered by some midwives, partly due to the unfamiliarity of the calibration process and, in part, due to malfunction of the device. Midwives were committed to troubleshooting these technical difficulties and spent considerable time attempting to address the malfunction—sometimes with success and other times without. In the event that continuing to use the beltless device proved not viable, the existing CTG technology remained readily available. This reduced anxiety amongst clinicians about adopting the NIFECG (PABS) technology, as familiar backup was available.

When the NIFECG (PABS) worked successfully, responses from women and clinicians were overwhelmingly positive, as demonstrated by a quote from one midwife who said ‘When it works I love it!’ When the device worked well throughout the first stage and/or first and second stages of labour, women and midwives felt very favourable towards its use. The reasons for this included comfort and freedom of movement for women and the lack of time needed to adjust the device (in comparison to the CTG) for midwives. When loss of contact (LOC) issues occurred, especially in relation to the fetal heart rate (FHR), they were not well tolerated by midwives and obstetricians. Despite this, some midwives liked the NIFECG (PABS) device so much; they stated it would be their first choice, even if it meant they had to swap to CTG in the event of LOC, as demonstrated by this quote:

‘I would try the [beltless device] first… Because if you can get it working, it just frees up the rest of your time… Every time they move, you're readjusting… So if it can free up those issues, then it's worth a shot every time’ (Midwife).

Upon receiving ethics and governance approval but prior to the commencement of the feasibility study, we piloted use of the NIFECG (PABS) device on five women at the study site. This provided reassurance to midwives and obstetricians that the interpretation of the visual data was consistent with existing practice, enhancing their readiness to adopt the new device. Qualitative data from obstetricians showed that most did not notice a difference in the visual data and did not feel that their practice had been impacted by use of the NIFECG. Not all feedback was positive, some obstetricians were frustrated by technical difficulties and felt that the device was not yet acceptable for adoption into clinical practice.

The clinicians were working in a high stress environment and participating women were classified as ‘high risk’. Hence, there was a low threshold for tolerating loss of contact with the fetal heart. Clinicians needed certainty that the device was accurately measuring the fetal and maternal heart rates and the woman’s uterine contractions. A low threshold for uncertainty meant that when the fetal heart rate showed signs of deviating from normal, clinicians were more likely to revert to familiar CTG technology. Inspection of the traces by the research team revealed that in many cases, reverting to CTG did not provide a more reliable trace than the NIFECG (PABS) had. However, it is unsurprising that clinicians felt more comfortable reverting to a known device when they felt uncertain about fetal wellbeing.

### Domain 5: The organisation

The study was carried out in a public women’s hospital—a multi-faceted, large hierarchical tertiary referral centre staffed by multidisciplinary clinicians providing care for women of both high and low acuity. The environment is busy, noisy and often high-stress. It integrates many different domains and models of care from low to high risk and forms part of a state-wide network offering care for complex women and babies.

The high level of senior managerial engagement and support for the study, especially from the lead obstetrician and CMC, was crucial to the successful conduct of the study. With support of the organisation, we undertook a comprehensive consultation process, including two lunchtime presentations about the device and plans for the study and multiple ward-based presentations to midwives. A study website was made available which included literature containing evidence about the device. The CMC drove recruitment and uptake of the study at the site. The CMC also engaged a research midwife, whose casual employment was financed by the study, to assist with recruitment, record keeping and liaison with midwifery staff. All women, midwives and obstetricians who were interested in participating were offered the opportunity to contact researchers by phone or email to ask questions. Part of this consultation process was to enable the co-design of data collection methods and to ensure a collaborative approach between the research team and clinicians.

This particular hospital’s track record of taking up new fetal monitoring technologies such as telemetry (wireless CTG) augured well for its likelihood to readily adopt this new technology. The Philips Avalon CTG is part of the infrastructure of the birth unit; hence, the beltless solution was perceived by clinicians as a minor upgrade to existing technology, not a completely new device. It is routine in this organisation for midwives to support women who are mobilising in labour; hence, there were few adaptations needed to their fundamental caregiving. The technology also aligns with the organisation’s core business of providing intrapartum care for women with complex pregnancies.

### Domain 6: The wider system

Policies and guidelines direct the use of continuous EFM in all maternity hospitals. In Australia, approximately 300,000 women give birth each year [10], and it is estimated that more than half experience continuous EFM, as described in the Background section. This is further driven by a lowering of the threshold for the indications for the use of continuous EFM, as evidenced by the changes in policy over the past decade that have seen increasing indications for its use [11, 34–36]. In Australia, it appears that this increase in use of continuous EFM may also be driven by medico-legal considerations and the need for documentary evidence of the fetal monitoring over the course of labour, in the event of adverse outcomes that result in legal proceedings.

Caesarean section rates in Australia are among the highest in the world. The most recent national data reported states 34% [10] and rates increase every year. High rates of caesarean section are of concern because unnecessary caesarean sections have significant short and long-term impacts upon the health of populations [37]. Enabling mobility in labour is a simple, cost-effective way to optimise physiological processes and strengthen women’s capabilities during their labour and birth, thereby reducing caesarean section rates.

In response to concerns about rising intervention rates, a significant international focus has developed in recent years on reversing these trends by promoting the facilitation of physiological processes and strategies such as mobility in labour and maternal choice of birth positioning [38–40]. A call to action by ‘an alliance of global stakeholders’ ([41] p2) including prominent midwifery academics from the UK, Europe, Australia and North America recently proposed a three-part approach to re-prioritise the research agenda in maternity care. The rationale for this study is underpinned by the second of those approaches, entitled ‘Research Priority B’ that aims to, ‘Identify and describe aspects of care that optimise, and those that disturb, the biological/physiological processes for healthy childbearing women and fetus/newborn infants and for those who experience complications’ ([41] p6).

There is growing international evidence on the relationship between women’s human rights and the right to informed consent, including the right to decline intervention. In the context of fetal monitoring, there is an urgent need to reflect on our relationship with technology so that physiological processes and positive experiences for women remain paramount. One may ask, for example, at what other time in a woman’s life would it be considered reasonable to tether her to a machine that restricts her movement to a range of 1.5 metres?

### Domain 7: Embedding and adaptation over time

The key findings of this feasibility study are the positive responses from the majority of stakeholders in relation to the potential for this device to replace the CTG. The comfort afforded by the beltless design and the lightweight nature of the device was expressed by all women participants. Freedom of movement and positioning was enabled for women after the initial calibration period, which makes a significant contribution to optimising women’s bodily autonomy and physiological processes in labour. Midwives reported when no technical difficulties occurred, this device enabled them to focus less on the technology and more on supporting women’s physical and emotional needs during labour. Midwives and obstetricians expressed a need for greater certainty about the reliability of the signal.

There was a sense of uncertainty for clinicians about the device working reliably and consistently. Whilst the reliability in measuring uterine activity was relatively good (83% of cases), there was a sense of uncertainty around contact with the fetal heart rate. In 27.3% of cases, midwives were unable to get all three signals (fetal heart, maternal heart and uterine activity) to work, despite troubleshooting for 30–90 min. In 34 cases, the device worked well for a length of time but was later discontinued due to loss of contact with one or more signals. When the device was discontinued, the midwife was able to swap to use of the external CTG.

The implications of the feasibility findings for the design of the main study are that further work is needed to refine the reliability of the technology in relation to preventing unexpected loss of contact, particularly in second stage. Facilitators of the process of embedding and adapting over time include the openness of the manufacturer to consult with the CI regarding reliability, freedom of movement, the role of midwives in EFM and the clinical application of this technology.

## Summary/strengths and limitations

This feasibility study was conducted in an urban setting that has low rates of women with overweight and obesity. Due to the existing experimental evidence around the increased efficacy and effectiveness of NIFECG over the CTG in women with high BMI, further research is needed in this cohort in the Australian context. This is important because overweight and obesity affects approximately half the population of women of childbearing age (18–44 years of age) in Australia [1].

A strength of this project is that it is led by midwives, who are well placed to provide data on the impact to their practice. Midwives have expertise in and responsibility for obtaining information about fetal wellbeing and are dealing with uncertainty and decision making around this in their daily work. The majority of the clinical literature about fetal monitoring has traditionally been led by obstetricians, often without engagement with midwives or women. This study adds valuable insight into midwifery practice around the use of fetal monitoring technologies and midwives are well placed to understand and convey what is important to women in their childbirth experience.

The chief investigator met regularly via Skype with key staff from the manufacturer (Philips) in Germany and Australia, to discuss clinicians’ feedback on the device and this consultation is ongoing. This opportunity enables the midwifery profession to express their perspective on the needs of midwives and women and is a crucial contribution to the co-design of technologies that will impact the future of maternity care and women’s experiences of childbirth.

Future research will include an investigation of the effectiveness and feasibility of using the NIFECG (PABS) specifically for intrapartum fetal monitoring of women with increased BMI. Despite a plethora of experimental studies conducted on women during short periods (30–90 min) of labour, this study is one of the first to use a NIFECG technology in a real world setting for the entire duration of participating women’s labour and birth.

Limitations of this study include that it was conducted at a single tertiary hospital site in an urban area where local women are of a high socioeconomic status, well-educated and generally trusting of health services. Further research is needed to explore barriers and facilitators to implementation in different settings, particularly regional, rural and remote contexts.

Due to the need for women to sit quietly for 15 min to allow the device to calibrate, recruitment became targeted at women who would be admitted to hospital prior to active phase of labour. This meant that there was a high rate of inductions and hence a high rate of epidural anaesthesia. This may limit generalisability.

There is a potential for bias due to the involvement of the manufacturer; however, this was mitigated by the fact that the study was investigator designed and investigator led. No individuals from the technology company were included in the research team, nor did they have any input into the reporting or dissemination.

This is the first time the NASSS framework has been used to synthesise the implementation needs of a health technology in the care of women during labour and birth. The NASSS framework was found to be a very useful tool that encouraged the researchers to apply a range of micro, meso and macro perspectives to the analysis. The authors plan to enrich this knowledge by using the framework again in future research in different maternity care settings and with different cohorts of women and clinicians.

## Conclusion

A variety of quantitative and qualitative data collected were synthesised to address each of the seven domains of the NASSS framework. Following this analysis, the authors were able to reach a consensus that the condition of childbirth is complex and the technology complicated, whilst the other domains; value proposition, the adopters, the organisational setting of this study, the wider system and embedding and adaptation, may be classified as simple.

In organisations where freedom of movement and positioning is not routine and/or undervalued, it may be more complicated to embed the NIFECG. In enabling more women to mobilise whilst wearing a wireless and beltless device, midwifery practice will expand to its full scope in this area. In some settings, staff may be more comfortable caring for women only in limited positions on the obstetric bed. Adoption of a wireless technology may be slower in such settings due to wider implications for practice.

Our findings contribute new knowledge about the implementation process and about the determinants for implementation of a complex technology in a maternity care setting. The identified complexity levels of the different domains of the technical innovation provide valuable insights to guide implementation and scale up.

## Supplementary Information

**Additional file 1.** The Philips Avalon Beltless solution pictured here is attached to the woman’s abdomen using five small adhesive electrodes similar to those used for adult cardiac monitoring by electrocardiogram (ECG). Signals are transmitted via a small pod that is similar in size to a matchbox. Unlike the CTG, once applied, the wearable device should not require adjustment by the midwife when the woman mobilises or her fetus changes position. The beltless solution records and digitises data pertaining to the fetal heart rate, maternal heart rate and uterine activity, transmitting data wirelessly from the pod (attached to the woman’s abdomen) to the base station. Although different technology is used to collect the data versus conventional CTG monitoring, the base station is compatible with existing CTG machinery and infrastructure installed in the majority of Australian hospitals. No additional skills or knowledge are required from clinicians to interpret the visual data, which appear almost identical to the data produced by a CTG.

## Data Availability

The datasets generated and/or analysed during the current study are not publicly available as the sample is small and derived from one site, thereby presenting a risk to participants’ confidentiality. Aggregated data on outcomes are available from the corresponding author on reasonable request.
