# Supplementary material for: Harnessing technology to enable all women mobility in labour and birth: feasibility of implementing beltless non-invasive fetal ECG applying the NASSS framework
Source: Pilot Feasibility Stud. 2021 Dec 7;7:214. doi: 10.1186/s40814-021-00953-6 (PMC8650358; doi:10.1186/s40814-021-00953-6)

Appendix 1

The Philips Avalon Beltless solution (pictured below) is attached to the woman’s abdomen using five small adhesive electrodes similar to those used for adult cardiac monitoring by electrocardiogram (ECG). Signals are transmitted via a small pod that is similar in size to a matchbox. Unlike the CTG, once applied, the wearable device should not require adjustment by the midwife when the woman mobilises or her fetus changes position. The beltless solution records and digitises data pertaining to the fetal heart rate, maternal heart rate and uterine activity, transmitting data wirelessly from the pod (attached to the woman’s abdomen) to the base station. Although different technology is used to collect the data versus conventional CTG monitoring, the base station is compatible with existing CTG machinery and infrastructure installed in the majority of Australian hospitals. No additional skills or knowledge are required from clinicians to interpret the visual data, which appear almost identical to the data produced by a CTG.


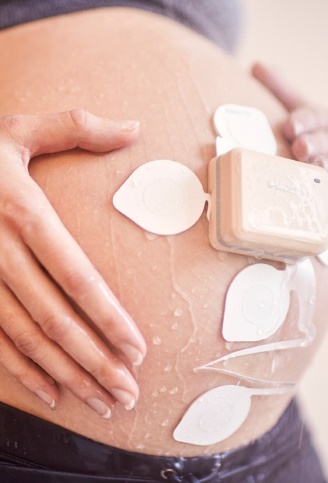

Supplement: Supplementary file 1 — Additional file 1. The Philips Avalon Beltless solution pictured here is attached to the woman’s abdomen using five small adhesive electrodes similar to those used for adult cardiac monitoring by electrocardiogram (ECG). Signals are transmitted via a small pod that is similar in size to a matchbox. Unlike the CTG, once applied, the wearable device should not require adjustment by the midwife when the woman mobilises or her fetus changes position. The beltless solution records and digitises data pertaining to the fetal heart rate, maternal heart rate and uterine activity, transmitting data wirelessly from the pod (attached to the woman’s abdomen) to the base station. Although different technology is used to collect the data versus conventional CTG monitoring, the base station is compatible with existing CTG machinery and infrastructure installed in the majority of Australian hospitals. No additional skills or knowledge are required from clinicians to interpret the visual data, which appear almost identical to the data produced by a CTG. [file 40814_2021_953_MOESM1_ESM.docx]
